# Comparison of the recurrence rate of different surgical techniques for oral mucocele: A systematic review and Meta-Analysis

**DOI:** 10.4317/medoral.26015

**Published:** 2023-06-18

**Authors:** Mohsen Hashemi, Mahsa Zohdi, Ehsan Zakeri, Tannaz Abdollahzadeh-Baghaei, Katayoun Katebi

**Affiliations:** 1 ORCID: 0000-0002-9145-8652. Department of Oral and Maxillofacial Surgery, Faculty of Dentistry, Tabriz University of Medical Sciences, Tabriz, Iran; 2 ORCID:0000-0001-5390-7712. Student Research Committee, Tabriz University of Medical Sciences, Tabriz, Iran; 3 ORCID: 0000-0003-3606-3671. Faculty of Dentistry, Tabriz University of Medical Sciences, Tabriz, Iran; 4 ORCID: 0000-0001-7767-9590. Department of Orthodontics, Faculty of Dentistry, Tabriz University of Medical Sciences, Tabriz, Iran; 5 ORCID: 0000-0002-6595-6359. Department of Oral and Maxillofacial Medicine, Faculty of Dentistry, Tabriz University of Medical Sciences, Tabriz, Iran

## Abstract

**Background:**

There are different surgical techniques to remove Oral mucoceles, including conventional surgery with scalpel, removal of the lesion with CO2 laser, and micro marsupialization. The present systematic review was conducted with the aim of comparing the recurrence rate of different surgical techniques for treatment of the oral mucoceles.

**Material and Methods:**

An electronic search for randomized controlled trials published in English until September 2022 related to different surgical methods for the treatment of oral mucocele was performed in Medline/PubMed, Web of Science, Scopus, Embase and Cochrane databases. A random-effects meta-analysis was conducted to compare the recurrence rate of different techniques.

**Results:**

Among 1204 papers initially identified, after the removal of duplicate articles and screening of the titles and abstracts, fourteen full-text articles were reviewed. Seven articles comparing the recurrence rate of oral mucocele in different surgical techniques were found. Seven studies were included in qualitative studies, and five articles were included in the meta-analysis. The risk of mucocele recurrence in the micro-marsupialization technique was 1.30 times that of the surgical excision with scalpel technique, which was not statistically significant. The risk of mucocele recurrence in the CO2 Laser Vaporization technique was 0.60 times that of the Surgical Excision with Scalpel technique, which was not statistically significant.

**Conclusions:**

The results of this systematic review showed that there is no significant difference between the recurrence rate of surgical excision, CO2 laser and marsupialization techniques for the treatment of oral mucoceles. Although more randomized clinical trials are needed for definitive results.

** Key words:**Mucocele, lasers, gas, recurrence, meta-analysis.

## Introduction

Oral mucocele is a cyst-like lesion and the second benign soft tissue lesion in the oral cavity, which is resulted from the accumulation of saliva caused by minor pathological changes in the salivary glands of the mouth (1). Clinically, it appears as single or multiple, soft, smooth, spherical nodules, without pain and different colors appear from clear blue to pink (2).

Oral mucoceles are asymptomatic in most cases, but in rare cases, when they appear as multiple and recurring lesions, they may cause severe pain (3). If the extension is large enough, it may interfere with speech as well as chewing and swallowing food. In some cases, this condition may be accompanied by excessive secretion of saliva into the mouth (4).

There are different surgical techniques to remove this lesion, including: conventional surgery with scalpel, removal of the lesion with CO2 laser, marsupialization and micro marsupialization. Among these, conventional scalpel surgery is the most common method for the treatment of this lesion despite complications such as postoperative bleeding and neuropathy, pain, lip deformity, damage to nearby anatomic structures and ducts (5).

Laser has many advantages over scalpel in soft tissue surgeries. Lasers can vaporize, coagulate, or cut. The laser causes immediate sterilization of the surgical wound, provides a bloodless surgery in most cases, and allows surgery without contact and consequently mechanical damage to the tissue (6). At present, the efficiency of the CO2 laser due to its high speed, minimal swelling, negligible damage to nearby tissues, and less pain is considered higher compared to other techniques (7). However, due to the high cost of laser generators and lack of experience with it, this technology has not been fully developed in some centers (8).

Micro-marsupialization is simple and fast technique and causes the least damage among the described management options. Although, it has been reported that large and deep mucoceles treated with micro marsupialization technique might show poor clinical results (9). Therefore, the success rate depends on the case selection for micro marsupialization. The recovery of the lesions, the recent history of trauma, whether the lesion is superficial or deep, and the size of the lesion should be taken into account carefully (10).

The present systematic review and meta-analysis was conducted with the aim of comparing the recurrence rate of different surgical techniques for treatment of the oral mucoceles.

## Material and Methods

In this systematic review, the principal question was formulated on the basis of the population, intervention, comparison, and outcome (PICO) approach, where “P” was patients with mucocele, “I” was conventional surgery, “C” was other surgical techniques including marsupialization and laser, and “O” was recurrence rate. The aim of this study was to compare the recurrence rate of oral mucocele in different surgical techniques.

- Inclusion Criteria

Cross-sectional and Randomized Clinical Trials; Only studies focusing on patients with oral mucocele; Only studies using surgical techniques as a treatment; Only English papers; and Papers published until December 2022.

- Databases and Search Strategy

The article selection process was performed in five steps according to PRISMA flow diagram (11). The electronic search was conducted by KK.

Medline/PubMed, Web of Science, Scopus, Embase, and Cochrane databases were searched. The keywords were selected from medical subject heading terms (MESH) and free terms. The search keywords were “recurrence”, “mucocele”, “oral mucocele”, “surgery”, “conventional surgery”, “scalpel”, “blade”, “laser”, “laser surgery”, “diode laser”, “CO2 laser”, “micro-marsupialization”, “marsupialization”, “recurrence”, “relapse”, “mucoceles”, “ranula”, “sialocele”, and “cold knife”, “cold knife surgery”, “excision”, “diode laser”, “cryotherapy”, “laser therapy”.

Every possible combination of free and medical subject heading terms with “OR” and “AND” operators was considered for finding the data. The research team made an effort to communicate with the Correspondences for supplementary information if necessary. To identify more research studies, the reference lists of the selected studies were searched as well.

The EndNote Basic software was used to manage the references, and duplicate references were identified and removed.

- Study Selection

Two reviewers (K.K and M.Z) scanned the titles and abstracts of the articles independently. In the second step, the full texts of the selected articles were reviewed. In the case of a disagreement, a third reviewer (M.H) was consulted. Finally, the full-text evaluation of the included articles was performed using a pre-designed data extraction form.

- Assessment of the Risk of Bias

The Cochrane risk-of-bias tool for RCTs version 2 (RoB2) (12) was utilized by two independent reviewers (K.K and E.Z) to appraise the selected article. Disagreements were settled by discussing with a third reviewer (M.H). Articles with high risk of bias, including studies without a control group were excluded from the study.

- Data Collection Process

A customized form for data extraction was built in Microsoft Excel to classify the details of the studies, including study ID (first author’s name and publication date), country of origin, surgical methods used, genders, mean age, mean size of lesion, location, follow up time, sample size, recurrence rate, and complications.

- Statistical Analysis

The mean recurrence rate with standard deviation was calculated for the included studies. Heterogeneity between studies was calculated using the I2 and Q indices. A value of I2 greater than 50% was considered significant heterogeneity. For combining the results, a random-effects model was utilized. The Statistical analysis were performed using the CMA v.2.0 software. A probability value <0.05 was considered significant. Finally, the results of the meta-analysis were presented in the form of forest plots.

## Results

- Search Results

**Figure 1:**
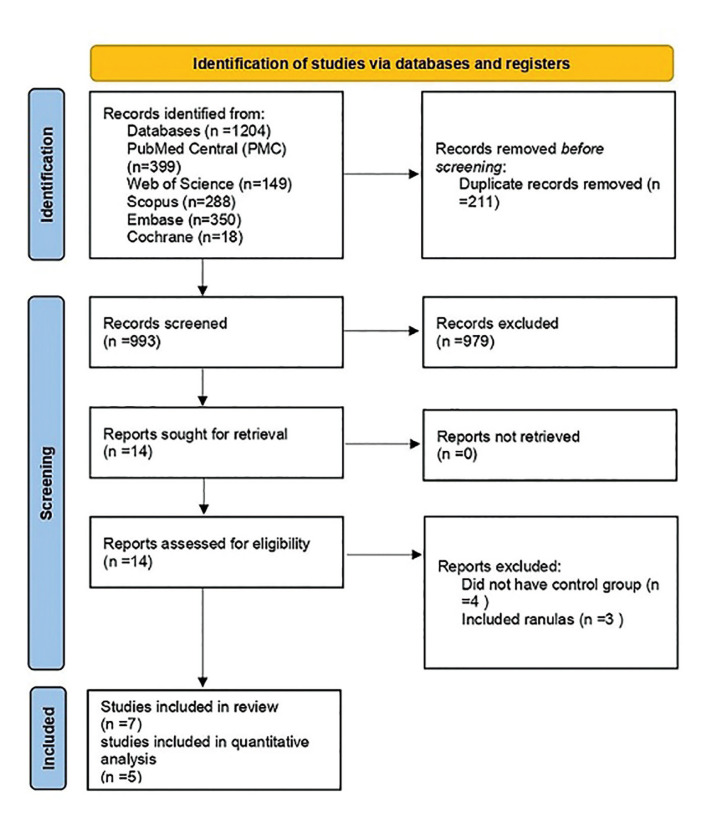
PRISMA flow diagram for the selection of eligible articles included in the study.

Among 1204 papers initially identified, 993 studies remained to be assessed after the removal of duplicate articles. After an initial screening of the titles and abstracts, fourteen full-text articles were reviewed (Fig. 1).

According to the inclusion and exclusion criteria, seven articles were found that compared the recurrence rate of oral mucocele in different surgical techniques. Therefore, seven studies were included in qualitative studies (13-19), and five were included in the meta-analysis (13-17).

- The Results of Evaluating the Risk of Bias

**Figure 2:**
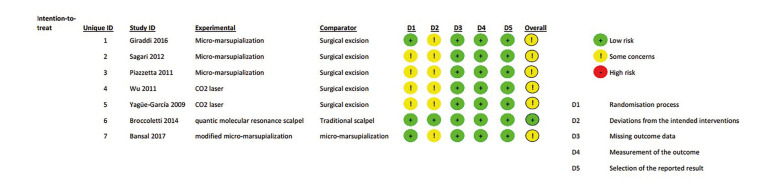
Risk of bias assessment.

None of the articles showed high risk of bias. According to RoB2, one of the studies showed a low risk of bias (18), while the other 6 had moderate risk of bias (13-17,19). The details are presented in Fig. 2.

- Characteristics of the Studies

Seven studies were included comprising of 368 patients. The descriptive characteristics and the associated data of these studies are organized in Table 1. Follow up periods ranged from one months to twelve months. Recurrence rate in the studies ranged from 0% to 42%.

Three studies compared micro-marsupialization and surgical excision (13-15). Overall, 51 patients were treated with micro-marsupialization and 70 patients were treated with surgical excision.

**Table 1:**
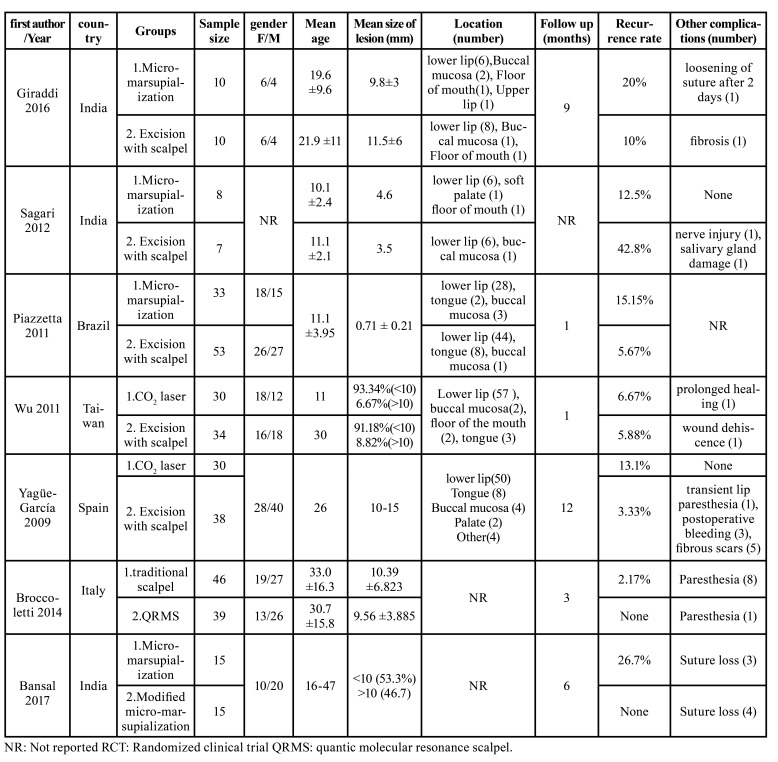
The data of the studies included in the systematic review.

Two of the studies reported higher rate of recurrence in micro-marsupialization method but one of the studies’ results was different. In spite of the results, all three studies reported that the difference was not statistically significant.

Two studies compared Mucocele removal by CO2 laser and surgical excision (16,17). In the study done by Wu *et al*. four (6.25%) recurrences happened, two of which were from the CO2 laser group (6.67%) and the other two were from the surgical excision group (5.88%) (16). In the other hand, Yagüe-García *et al*. reported that ablation of the oral mucoceles with the CO2 laser resulted in more predicTable outcomes and the frequency of complications and recurrences were lower than scalpel surgery (17).

Bansal *et al*. (19) compared micro-marsupialization with modified micro-marsupialization. Modified micro- marsupialization uses maximum possible number of sutures with a short distance between entry and exit. No recurrences were reported in the first month. After three months, one case, and after six months, three cases, had recurrences from micro-marsupialization group. However, the difference between groups was not significant.

In a study conducted by Broccoletti *et al*. (18) traditional or quantic molecular resonance scalpel surgery (QMRS) were used for eighty-five patients with mucoceles. After three months of follow-up, one recurrence from the cold scalpel group was reported. No recurrence happened in QMRS group. No significant difference was found between the two surgical techniques. The average painkiller use in the cold scalpel and QMRS groups was 1.46 and 1.56, respectively (*p*>.05).

- Meta-Analysis

In three studies (13-15) the recurrence rate of oral mucocele was investigated between the two techniques of surgical excision with scalpel and micro-marsupialization. There were 70 patients in the surgical excision with scalpel group and 51 people underwent micro-marsupialization.

**Figure 3:**
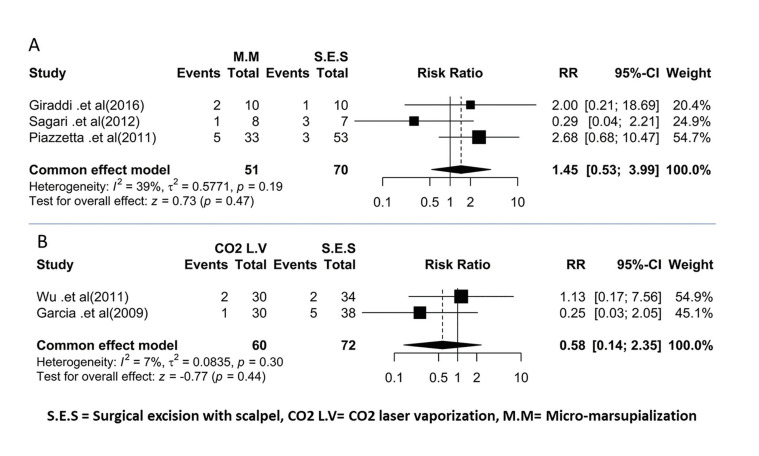
A) Forest plot of the comparison between micro-marsupialization and Surgical excision. B) Forest plot of the comparison between Co2 laser and Surgical excision.

The heterogenicity between studies was not significant (Q-value=3.261, df=2, I-square=38.66, *p-value*=0.196). Based on the results of the meta-analysis, the risk of mucocele recurrence in the micro-marsupialization technique was 1.30 times that of the surgical excision with scalpel, which is not statistically significant (Pooled RR=1.30, 95% CI=0.34-5.02, z- value=0.38, *p-value*=0.70). Fig. 3 shows the forest plot of the results of the meta-analysis.

In two studies (16,17), the recurrence rate of oral mucocele was investigated between two techniques, Surgical Excision with Scalpel and CO2 Laser Vaporization. 72 patients underwent surgical excision with scalpel and 60 underwent CO2 Laser vaporization. The heterogenicity between studies was not significant (Q-value=0.919, df=1, I-square=0.000, *p-value*=0.338). Based on the results of the meta-analysis, the risk of mucocele recurrence in the CO2 Laser vaporization technique was 0.60 times that of the Surgical Excision with Scalpel technique, which was not statistically significant (Pooled RR=0.60, 95% CI=0.15-2.40, z- value=-0.72, *p-value*=0.47). Fig. 3 shows the forest plot resulting from the meta-analysis.

**Figure 4:**
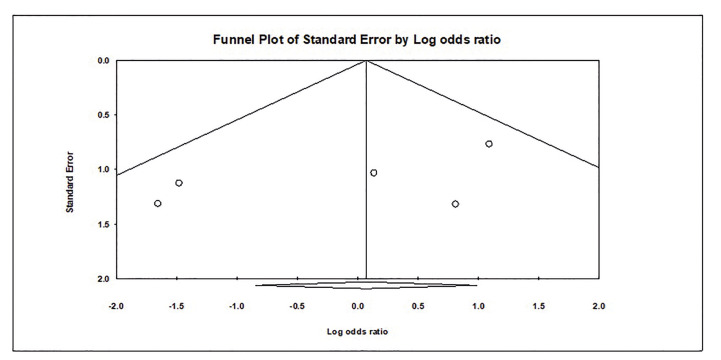
Funnel plot of the included studies.

Fig. 4 shows the Funnel diagram to check the Publication bias. Based on the presence of symmetry in Funnel plot and Egger's regression test, the publication bias was not statistically significant among the studies included in the study (t-value=1.50, df=3, *p-value*=0.23).

## Discussion

The present study reviewed the recurrence rate of oral mucocele in different surgical methods.

Although oral mucoceles may rupture spontaneously and heal without treatment within four to six weeks, recurrence is common (20). Some interventions may be needed to eliminate the large mucoceles (21). Although surgical excision is the most commonly used intervention to remove these lesions, other methods include marsupialization, micro-marsupialization, CO2 laser ablation, cryotherapy, intralesional steroid injection, and the use of sclerosing agents (22).

Conventional surgical excision which removes the adjacent small salivary ducts is the most common treatment of small mucoceles (23). On the other hand, this conventional surgery may damage the surrounding structures, especially the labial branch of the mental nerve in the case of large mucoceles, therefore marsupialization and other techniques were suggested (24,25). Micro-marsupialization which is a popular option for pediatric patients, is minimally invasive, fast, simple to perform, and relatively painless (26).

Among the included studies, three studies compared micro-marsupialization with surgical excision (13-15). Sagari *et al*. (15) reported that in 15 cases, three cases had recurrence in the surgical excision group, whereas in micro-marsupialization group, one case of recurrence was seen. There was no statistically significant difference between the groups in lesion evolution, recurrence or post-operative healing. Piazzetta *et al*. (14) reported that from the thirty-three cases treated by micro-marsupialization, twenty-five had a full recovery in seven days and three had full regression in fifteen days. In the other five cases, the lesions recurred and surgical excision was performed. In the surgical excision group, three of the fifty-three patients had a recurrence and second surgical excision was warranted. The rate of recurrence was not statistically different between the two groups. It was shown that mucoceles that had been present for less than ninety days responded better to micro-marsupialization compared to longer lasting mucoceles. In the study conducted by Giraddi *et al*. (13) in the group treated with micro-marsupialization two patients had a recurrence while in patients treated with surgical excision, there was one recurrence. All recurrent cases received a surgical excision. No statistically significant difference was found between the two groups.

All three studies reported that there was no statistically significant difference between the recurrence rates of these two methods. In addition, heterogenicity between studies was not significant and our meta-analysis showed no significant difference.

Bansal *et al* (19) assessed micro-marsupialization and marsupialization and declared no significant difference between recurrence rates. Modified micro-marsupialization appears to be a safe method of treatment for oral mucoceles. Many studies have mentioned the strengths of this modified method like being minimally invasive , shorter procedural time making it valuable in children, negligeable discomfort, and nearly no postoperative complications (27,28).

Lip disFigurement, damage to nearby ducts, numbness, and scarring are complications related to conventional surgical excision (17,21). CO2 laser ablation was proposed as an alternative treatment (29). CO2 laser ablation is a relatively fast and simple method which is limited to the superficial mucosa when set between 5 and 10 W. CO2 laser ablation results in fewer complications such as postoperative bleeding, pain, and damage to surrounding structures compared with scalpel excision (29,30). CO2 laser ablation does not require a suture and typically lasts about three to five minutes. It has excellent surgical visibility due to its bloodless procedure (31).

Among the studies that were reviewed, two studies compared CO2 laser with surgical excision with a Scalpel (16,17). In Wu *et al*. study,(16) 30 cases received CO2 laser and 34 received surgical excision. Two recurrences were reported from the CO2 laser group (6.67%) and two from the excision group (5.88%). Wound dehiscence happened in one case of the excision group and prolonged wound healing was seen in two patients from the CO2 laser group. The recurrence rate was not statistically different between the groups.

Yagüe-García (17) declared that among the conventional surgical removal group, 8.8% showed a recurrence, and 13.2% of the patients experienced postoperative complications. The most common complication was the presence of fibrous scars. There were no complications or recurrences after twelve months in the CO2 laser group.

This meta-analysis shows that the risk of recurrence of oral mucocele in patients treated with CO2 laser vaporization and surgical excision with a scalpel is not statistically different.

In one of the studies reviewed, Broccoletti *et al*. (18) concluded that QMRS for surgical removal of labial mucoceles was similar to traditional scalpel regarding postoperative quality of life, pain, and post-surgical lip paresthesia. QMRS uses electric current to produce a flux of quanta, therefore, the operator can cut and coagulate the tissue at the same time (32).

In general, several factors may affect the results of treatments, including age, gender, duration of follow-up, size of the lesion, location, presence of systemic diseases, and methods of post-operative care.

The limitation of this study is that it only evaluates the recurrence rate of the surgical methods not the other aspects of treatment outcomes or other non-surgical treatments.

## Conclusions

The results of this systematic review and meta-analysis showed that there is no significant difference between the recurrence rate of surgical excision, CO2 laser and marsupialization techniques for the treatment of oral mucoceles. Therefore, for now it can be said that the clinician should choose the method of treatment based on the availability of equipment and expertise, although more randomized clinical trials are needed for definitive results.
